# Association Between the Growth Hormone/Insulin-Like Growth Factor-1 Axis and Muscle Density in Children and Adolescents of Short Stature

**DOI:** 10.3389/fendo.2022.920200

**Published:** 2022-06-14

**Authors:** Guangzhi Yang, Qing Yang, Yanying Li, Yanhong Zhang, Shuxiong Chen, Dongye He, Mei Zhang, Bo Ban, Fupeng Liu

**Affiliations:** ^1^Department of Endocrinology, Affiliated Hospital of Jining Medical University, Jining, China; ^2^Department of Nutrition, Affiliated Hospital of Jining Medical University, Jining, China

**Keywords:** short stature, muscle density, GH/IGF-1 axis, body composition, GDDSD study

## Abstract

**Objective:**

To evaluate the association between the growth hormone (GH)/insulin-like growth factor-1 (IGF-1) axis and muscle density in children and adolescents of short stature.

**Methods:**

Participants were children and adolescents of short stature hospitalized in the Affiliated Hospital of Jining Medical University between January 2020 and June 2021. All participants had CT scan images available. We performed an analysis of the images to calculate the muscle density or skeletal muscle attenuation (SMA), skeletal muscle index (SMI), and fat mass index (FMI). Bioelectrical impedance analysis (BIA) was used to ensure that chest CT is a credible way of evaluating body composition.

**Results:**

A total of 297 subjects were included with the mean age of 10.00 ± 3.42 years, mean height standard deviation score (SDS) of -2.51 ± 0.53, and mean IGF-1 SDS of -0.60 ± 1.07. The areas of muscle and fat tissues at the fourth thoracic vertebra level in the CT images showed strong correlation with the total weights of the participants (*R^2^* = 0.884 and 0.897, respectively). The peak of GH was negatively associated with FMI (r = - 0.323, P <.01) and IGF-1 SDS was positively associated with SMI (r = 0.303, P <.01). Both the peak GH and IGF-1 SDS were positively associated with SMA (r = 0.244, P <.01 and r = 0.165, P <.05, respectively). Multiple stepwise linear regression analysis demonstrated that the GH peak was the predictor of FMI (β = - 0.210, P < .01), the IGF-1 SDS was the predictor of SMI (β = 0.224, P < .01), and both the peak GH and IGF-1 SDS were predictors of SMA (β = 0.180, P < .01 and β = 0.222, P < .01).

**Conclusions:**

A chest CT scan is a credible method of evaluating body composition in children and adolescents of short stature. In these patients, peak GH and IGF-1 SDS are independent predictors of muscle density and the GF/IGF-1 axis may regulate body composition through complex mechanisms.

## Introduction

Growth hormone (GH) promotes linear growth and plays key role in regulating muscle development and metabolism. Insulin-like growth factor-1 (IGF-1) is the major mediator by which GH elicits skeletal muscle cell proliferation and myocyte differentiation (1, 2). Children and adolescents of short stature often have increased fat mass and reduced lean mass and muscle strength. This phenomenon is more distinct in those with severe growth hormone deficiency (GHD) (3–5). Treatment with GH can increase muscle mass and strength and decrease fat tissue percentage. Discontinuing it leads to a reversal of these effects (4, 6–10).

Muscle density is an important parameter of muscle health and is emerging as a predictive factor for various metabolic diseases. In adults, low muscle density is associated with a high risk of diabetes, cardiovascular diseases, bone fractures, and worse outcomes in patients with cancer and other critical illnesses (11–18). Several studies have demonstrated that muscle density is a predictor of bone density, bone strength, and cardio-metabolic risk in children and adolescents (19–21). Currently, computed tomography (CT) is the gold standard for investigating qualitative changes in muscles. Low muscle attenuation indicates a high proportion of myosteatosis (intermuscular and intramuscular fat infiltration); whereas high muscle attenuation indicates low muscle fat infiltration (high muscle density) (16, 22, 23).

Although previous studies have reported that GH plays an important role in maintaining muscle mass, none have investigated the role of GH on muscle density. In the past two years, a chest CT scan was performed on some children and adolescents of short stature admitted to our hospital during the COVID-19 pandemic. We performed this retrospective study to evaluate the relationship between the GH/IGF-1 axis and muscle density in children and adolescents of short stature. The areas of skeletal muscle and fat at the fourth thoracic vertebra (T4), assessed by CT, and the total weight assessed by bioelectrical impedance analysis (BIA) were correlated to ensure chest CT was a credible method of evaluating body composition.

## Methods

### Study Patients

All the subjects enrolled were in the GDDSD study (http://www.chictr.org.cn, ChiCTR1900026510), an ongoing prospective, observational, open cohort study that is evaluating the etiology of growth and development diseases and the long-term safety and effectiveness of growth hormone therapy in a real-life clinical setting (24). Children and adolescents of short stature in the study were those hospitalized between January 2020 and June 2021 in the Department of Endocrinology of the Affiliated Hospital of Jining Medical University and had a chest CT scan done. Short stature is defined as a condition in which the individual’s height is two standard deviations (SD) or more below the population mean for the relevant age and gender (25). The exclusion criteria were as follows: (1) patients missing the values of IGF-1 and GH stimulation test; (2) patients with chronic disease, malignant tumors, and abnormal thyroid function; and (3) patients with conditions such as skeletal dysplasia, achondroplasia, and disorders of sex development. Approval was obtained from the Ethics Committee of the Affiliated Hospital of Jining Medical University and informed consent forms were signed by all the participants’ parents.

### Body Composition Measurements

Two authors of this study identified axial CT images at the T4 level and used them to calculate the skeletal muscle area, subcutaneous fat area, and mean skeletal muscle attenuation (SMA). The Slice-O-Matic software (V.5.0, TomoVision, Montreal, Quebec, Canada) was used in this analysis and the attenuation threshold was set to −29 to 150 Hounsfield units (HU) for skeletal muscle, and −190 to −30 HU for subcutaneous adipose tissue. Each type of tissue found in the T4 CT images was shaded with a different color that corresponded to these thresholds. The T4 cross-sectional skeletal muscle area (T4MA) and subcutaneous fat area (T4FA) were recorded in cm^2^ and the SMA in mean HU. The skeletal muscle index (SMI) and fat mass index (FMI) were calculated by dividing the skeletal muscle and subcutaneous fat area in cm^2^ by height in m^2^. The total muscle mass (TMW) and total fat tissue mass (TFW) were measured using BIA with patients in a fasting state.

### Laboratory Measurements

Overnight fasting blood samples were collected from all participants and laboratory parameters were measured using methods described in a study we did previously (24). The biochemical and immune indices used in this study include GH, IGF-1, IGF-binding protein-3 (IGFBP-3), hemoglobin (Hb), alanine aminotransferase (ALT), albumin (ALB), creatinine (Cr), triglycerides (TG), total cholesterol (TC), high-density lipoprotein (HDL-C), low-density lipoprotein (LDL-C), blood calcium (Ca), blood phosphate (P), free triiodothyronine (FT3), free thyroxine (FT4), thyroid-stimulating hormone (TSH), luteinizing hormone (LH) and follicle-stimulating hormone (FSH). The IGF-1 SD score (SDS) was calculated using the reference values in healthy children of the same age and sex (26). Two of three GH stimulating tests were performed to evaluate the peak level of GH (levodopa, 500 mg for those ≥ 30 kg, 250 mg for those < 30 kg and ≥ 15 kg, and 125 mg for those < 15 kg; insulin, 0.1-0.15 U/kg; and arginine, 0.5 mg/kg). Blood samples were collected at 0, 30, 60, 90, and 120 minutes to obtain serum GH concentrations at each of these points.

### Statistical Analysis

Continuous variables were summarized using the median and IQR for non-normally distributed data and the mean ± SD for normally distributed data. Categorical variables were summarized as the frequency count in percentage. Correlations between variables were assessed by Pearson’s correlation coefficient. Multiple stepwise linear regression analysis was used to identify independent factors associated with muscle density. Statistical analysis was performed using SPSS software (26.0; IBM, Armonk, NY). A *P* value less than 0.05 was considered statistically significant.

## Results

A total of 297 eligible participants (189 males and 108 females) were included. The mean age was 10.00 ± 3.42 years (ranging from 4 to 16 years). Their mean height SDS was −2.51 ± 0.53 and the mean IGF-1 SDS was -0.60 ± 1.07. The body compositions measured by BIA and chest CT and other parameters are summarized in **Table 1**.

**Table 1:** Characteristics of patients included in this study.

Characteristic	Patients	Value	Characteristic	Patients	Value
Age (years)	297	10.00 ± 3.42	Hb (g/L)	296	131.30 ± 11.04
Bone age (years)	289	8.43 ± 3.83	ALT (U/L)	296	14.70 ± 8.07
Peak of GH (ng/mL)	297	7.29 ± 5.00	ALB (g/L)	296	46.88 ± 2.86
IGF-1 SDS	297	-0.60 ± 1.07	Cr (umol/L)	295	43.82 ± 11.66
IGFBP-3 (μg/mL)	293	4.55 ± 1.31	TG (mmol/L)	282	0.68 (0.53,0.91)
High SDS	297	-2.51 ± 0.53	TC (mmol/L)	282	3.91 ± 0.75
Weight (kg)	297	27.94 ± 11.91	HDL (mmol/L)	282	1.52 ± 0.41
BMI SDS	297	-0.22 ± 1.53	LDL (mmol/L)	282	2.21 ± 0.52
SMA (HU)	297	47.54 ± 4.05	Ca (mmol/L)	296	2.45 ± 0.10
T4MA (cm^2^)	297	94.85 ± 31.63	P (mmol/L)	296	1.60 ± 0.16
T4FA (cm^2^)	297	33.83 (22.57,58.23)	FT3 (pmol/L)	294	6.88 ± 2.26
SMI (cm^2^/m^2^)	297	59.03 ± 8.83	FT4 (pmol/L)	294	17.90 ± 3.01
FMI (cm^2^/m^2^)	297	23.99 (15.59,37.22)	TSH (mIU/L)	294	2.66 ± 1.36
TMW (kg)	205	10.88 ± 4.64	LH (mIU/ml)	281	0.23 (0.01,1.57)
TFW (kg)	205	4.90 (2.90,7.95)	FSH (mIU/ml)	281	2.45 (1.05,4.36)

### The Relationship Between CT Scan and BIA in Measuring Body Composition

**Figure 1:**
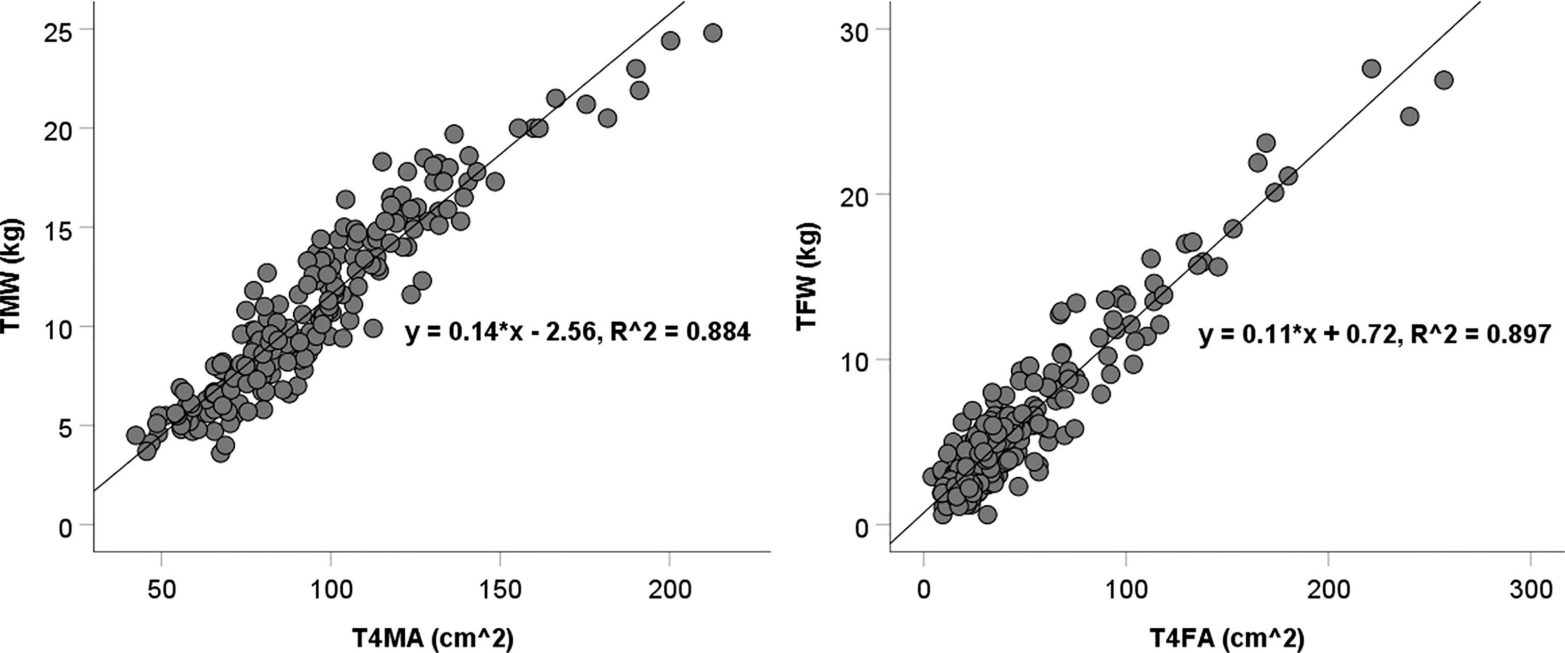
Scatter diagram of T4MA and T4FA with TMW and TFW. TMW, total muscle weight; TFW, total fat weight; T4MA, T4 muscle area; T4FA, T4 fat area.

Scatter plots show the T4MA and T4FA assessed by chest CT and TMW and TFW assessed by BIA (**Figure 1**). The T4MA showed a strong correlation with TMW (TMW = 0.14 T4MA – 2.56; *R^2^* = 0.884; *P* <.001) and T4FA showed a strong correlation with TFW (TFW = 0.11 T4FA + 0.72; *R^2^* = 0.897; *P* <.001).

### The Correlation Between the GH/IGF-1 Axis and CT Scan Body Composition

Male patients had a higher level of SMI (60.48 ± 8.93 and 56.79 ± 8.10, *P* <.01) compared with female patients, but there were no differences in FMI and SMA. The peak GH was negatively associated with FMI (r = - 0.323, *P* <.01) and positively associated with SMA (r = 0.244, *P* <.01). The level of IGF-1 SDS was positively associated with SMI (r = 0.303, *P* <.01) and SMA (r = 0.165, *P* <.05). The correlations between body composition and other clinical factors are shown in **Table 2**.

**Table 2:** Correlations between body composition evaluated by T4 CT scan and clinical factors.

Variables	SMI (cm^2^/m^2^)	FMI (cm^2^/m^2^)	SMA (HU)
Age (years)	- 0.027	0.195*	- 0.229**
Bone age (years)	0.036	0.234**	- 0.217**
High SDS	0.028	- 0.091	0.108
Weight (kg)	0.198*	0.535**	- 0.338**
BMI SDS	0.293**	0.563**	- 0.222**
Peak of GH (ng/mL)	0.071	- 0.323**	0.244**
IGF-1 SDS	0.303**	0.069	0.165*
IGFBP-3 (μg/mL)	0.054	0.132*	- 0.085
Hb (g/L)	0.216**	0.098	0.075
ALT (U/L)	0.101	0.404**	- 0.222**
ALB (g/L)	0.067	0.137*	- 0.060
Cr (μmol/L)	0.177*	0.053	0.073
TG (mmol/L)	0.135*	0.210**	0.005
TC (mmol/L)	- 0.144*	0.080	- 0.129*
HDL (mmol/L)	- 0.005	- 0.076	0.002
LDL (mmol/L)	- 0.026	0.289**	- 0.114
Ca (mmol/L)	0.049	0.112	- 0.042
P (mmol/L)	- 0.057	- 0.043	0.078
FT3 (pmol/L)	0.127*	0.136*	- 0.041
FT4 (pmol/L)	- 0.013	- 0.105	0.039
TSH (mIU/L)	0.029	0.058	0.004
LH (mIU/mL)	0.055	0.124*	- 0.103
FSH (mIU/mL)	0.008	0.214**	- 0.175*

Correlations are shown with the coefficient r value. * P < 0.05; ** P < 0.01.

Multiple stepwise linear regression analyses of variables related to the SMI, FMI and SMA are listed in **Table 3**. After adjusting for confounding factors, the peak GH was the predictor of FMI (β = - 0.210, *P* < .01) and SMA (β = 0.180, *P* < .01); IGF-1 SDS was the predictor of SMI (β = 0.224, *P* < 0.01) and SMA (β = 0.222, *P* < .01).

**Table 3:** Multiple stepwise linear regression analysis of factors associated with SMI, FMI and SMA.

SMI (cm^2^/m^2^)		FMI (cm^2^/m^2^)		SMA (HU)	
Variables	β value	Variables	β value	Variables	β value
IGF-1SDS	0.224**	BMI SDS	0.403**	Peak of GH (ng/ml)	0.180**
BMI SDS	0.278**	ALT (U/L)	0.234**	Age (years)	- 0.264**
Gender (male)	0.210**	Peak of GH (ng/mL)	- 0.210**	IGF-1SDS	0.222**
TC (mmol/L)	- 0.110*	LDL (mmol/L)	0.203**	BMI SDS	- 0.186**
–	–	Bone age (years)	0.186**	TC (mmol/L)	- 0.132*
–	–	FSH (mIU/mL)	0.109*	ALT (U/L)	- 0.134*
-	-	-	-	-	-

Adopted factors: gender, BMI SDS, IGF-1SDS, Hb, Cr, TG, TC and FT3 for SMI; age, bone age, BMI SDS, the peak of GH, IGFBP-3, ALT, ALB, TG, LDL, FT3, LH and FSH for FMI; age, bone age, BMI SDS, the peak of GH, IGF-1SDS, ALT, TC and FSH for SMA. * P < 0.05; ** P < 0.01.

## Discussion

In this study, the peak GH and IGF-1 SDS are positively correlated with muscle density in children and adolescents of short stature. After adjusting for confounding factors, both the peak GH and IGF-1 SDS are independent predictors of muscle density. To the best of our knowledge, this is the first study that demonstrates the relationship between the GH/IGF-1 axis and muscle density.

Several technologies including ultrasonography, dual x-ray absorptiometry, BIA, CT, and magnetic resonance imaging could be used to assess body composition (27). Among these, CT scans quantify bone mineral density, visceral and subcutaneous fat, skeletal muscle, liver fat, and arterial vascular calcification. Thus, they are the most comprehensive modality (28). The predictive value of a chest CT in whole-body composition has been evaluated in healthy adults or patients with cancer. The cross-sectional areas of muscle and fat tissue have shown a moderate correlation with total body weight (29–31). In this study, most of the participants are prepubertal or adolescent, and there is little interference from the abdomen, hip, and limbs. Our research demonstrated that the areas of muscle and fat tissues at the T4 level assessed by chest CT highly correlated with the total weights assessed by BIA (*R^2^* = 0.884 and 0.897, respectively). This provides a possibility of assessing body composition incidentally in some children if chest CT is required for their diagnosis and treatment.

Children and adolescents of short stature, especially those with GHD, often have increased fat mass and reduced lean mass and muscle strength. In 2016, Improda et al. summarized the role of the GH/IGF-1 axis in the muscle and skeletal health of children and adolescents (32). In general, childhood-onset GHD can affect bone and muscle mass and strength, and GH replacement therapy has beneficial effects. Moreover, GH withdrawal at final height can result in reduced bone and muscle mass, potentially leading to increased fracture risk in adulthood (32). Our study also confirmed the association between the GH/IGF-1 axis and body composition. The peak GH is correlated with FMI and IGF-1 SDS is correlated with SMI. This indicates that the GH/IGF-1 axis uses different mechanisms in the regulation of muscle and fat development and metabolism. Unlike skeletal muscle cell proliferation and myocyte differentiation by GH, which are almost entirely mediated by IGF-1, adipose tissue lipolysis appears to be directly mediated *via* the GH receptor (33–35). The body composition, in turn, also affects the levels of the peak GH and IGF-1. For example, obesity reversibly suppresses GH secretion driven by elevated free fatty acids, whereas IGF-I levels remain normal or elevated due to elevated portal insulin levels (36). Therefore, there are bidirectional associations exist between the GH/IGF-1 axis and body composition.

It is well known that adults of short stature or GHD are at a higher risk of hypertension, dyslipidemia, cardiovascular disease, type 2 diabetes, and fracture (37–42). Coincidentally, individuals with lower muscle attenuation on CT also have a higher risk of these metabolic diseases (15, 16, 43–47). It is possible that patients of short stature already have impaired muscle density in their childhood and the GH/IGF-1 axis plays a critical role in muscle density regulation. Our study demonstrated that both the peak GH and IGF-1 SDS are independent predictors of SMA in children and adolescents of short stature. SMA is a comprehensive marker that determines both SMI and FMI, and the GH/IGF-1 axis may regulate muscle density through complex mechanisms (48, 49).

Our study has several limitations. First, the cross-sectional design of the study does not allow for causal inference and is limited in clarifying the underlying pathophysiological mechanisms involved. Second, to determine whether lower muscle density in childhood can predict higher risks of metabolic diseases, a long-term follow-up is required. Third, some confounding factors that might influence muscle health such as family income, exercise intensity, and dietary habits were not included in the analysis. Fourth, most of the participants enrolled in our study were prepubertal and only SMI showed a gender difference in them. We did not perform subgroup analyses like those done in adult studies. Lastly, our study was conducted in China and the findings may not be readily generalizable in other populations or ethnicities.

In conclusion, this study confirmed the credibility of chest CT in evaluating body composition in children and adolescents of short stature. In these patients, both the peak GH and IGF-1 SDS are independent predictors of muscle density; and the GH/IGF-1 axis may regulate body composition through complex mechanisms.

## Data Availability Statement

The original contributions presented in the study are included in the article/**Supplementary Material**. Further inquiries can be directed to the corresponding authors.

## Ethics Statement

The studies involving human participants were reviewed and approved by The Ethics Committee of the Affiliated Hospital of Jining Medical University (2019C003, Jining, China). Written informed consent to participate in this study was provided by the participants’ legal guardian/next of kin.

## Author Contributions

The manuscript was conceived by QY and FL, with manuscript questions and analytic plan designed by GY, QY, BB, and FL. FL wrote the manuscript, interpreted the data, critically reviewed and revised the manuscript. GY, QY, MZ, and BB contributed to writing, data analysis, data interpretation, critical review and revision. YL, YZ, SC, and DH contributed to data interpretation, critical review and revision. All authors had access to the data and all authors agreed to submit the final manuscript. FL was supported by the Jining Key Research and Development Projects. GY, QY, and FL are the guarantors of this work and as such had full access to all the data in the study and take responsibility for the integrity of the data and the accuracy of the data analysis.

## Funding

The study was supported by the Jining Key Research and Development Projects (2021YXNS073).

## Conflict of Interest

The authors declare that the research was conducted in the absence of any commercial or financial relationships that could be construed as a potential conflict of interest.

## Publisher’s Note

All claims expressed in this article are solely those of the authors and do not necessarily represent those of their affiliated organizations, or those of the publisher, the editors and the reviewers. Any product that may be evaluated in this article, or claim that may be made by its manufacturer, is not guaranteed or endorsed by the publisher.
